# Editorial: New insights in the neuroanatomy and neuropathology of marine mammals

**DOI:** 10.3389/fnana.2024.1449199

**Published:** 2024-07-01

**Authors:** Simona Sacchini, Cristiano Bombardi

**Affiliations:** ^1^Department of Morphology, Campus Universitario de San Cristóbal, University of Las Palmas de Gran Canaria, Las Palmas de Gran Canaria, Spain; ^2^Department of Veterinary Medical Science, University of Bologna, Bologna, Italy

**Keywords:** neuroanatomy, neuropathology, neurodegenerative diseases, marine mammals, cetaceans, toothed whales, planetary health

Cetaceans in particular, as well as other marine animals in general, offer important insights about the health of our oceans. Among cetaceans, toothed whales are consequently considered bioindicator species and sentinels of the sea's health, in a concept known as “planetary health”. Furthermore, these marine animals provide new and more realistic comparative natural models for the study of particular brain problems (i.e., neurodegenerative diseases) because of their longevity, which is similar to that of humans. Yet, many concerns remain in the fields of neuroanatomy and neurohistology related to marine mammals and thus cetaceans. Actually, the majority of research focuses on the cerebral cortex and is restricted to a few species, leaving out those whose brain samples are more challenging to collect, as in the case of beaked whales. In fact, cytoarchitecture of the visual and auditory cortex, cortical thickness, cerebellar mass, cortex surface area, and neuron density values are among the few neurohistological data known concerning the beaked whales brain. To improve the interpretation of neuropathological changes and their etiopathogenesis, as well to understand age-related changes, a solid understanding of neuroanatomical structures is required. Regarding advances in this Research Topic, Graïc et al. provided novel insights on two significant aspects of the bottlenose dolphin's (*Tursiops truncatus*, family *Delphinidae*) brain: the characterization of the entorhinal cortex and age-related changes in the primary auditory cortex. Graïc, Grandis et al. explain how calcium-binding proteins (namely calbindin D-28k, calretinin and parvalbumin) are expressed by neurons that have distinct morphological classes in the entorhinal cortex. Since it is a target region in Alzheimer's disease (AD), the entorhinal cortex is an excellent sample area in routine neuropathological analysis. AD involves predominantly cerebral cortex, as well as subcortical and brainstem areas, with β-amyloid deposition and hyperphosphorylated tau in the form of neurofibrillary tangles (Schröder et al., 2023). The distribution pattern of neurofibrillary pathological alterations serves as the basis for the Braak staging, as follows: first entorhinal cortex (Braak I and II), followed by the limbic (Braak III and IV), and finally the isocortical (Braak V and VI). The entorhinal cortex has only been recently explored in a beached harbor porpoise (*Phocoena phocoena*; Family *Phocoenidae*), exposed to cyanobacterial β- N-methylamino-L-alanine (BMAA) toxin (Garamszegi et al., 2024). After the animal's brain was examined for signs of AD-like disease, Aβ+ plaques were found in the entorhinal cortex and the amygdaloid complex along with other neurodegenerative alterations. If we move to other marine mammals, a study conducted on 8 aged pinniped species, explored the entorhinal cortex in 2 out of them. Interestingly, the 2 animals, a California sea lion (*Zalophus californianus*, family *Otariidae*) and an Australian sea lion (*Neophoca cinerea*, family *Otariidae*), both aged 32, showed severe lesions of AD-like pathology affecting also the entorhinal cortex (Takaichi et al., 2021). On the other side, Graïc, Corain et al. reported in the auditory cortex a seemingly decreasing surface density in elderly animals compared to adults. Moreover, thin and bigger neurons were also described in elderly animals and, according to the authors, may be consistent with the accumulation of tau protein or lipofuscin. Beyond the neuropathological conclusions that can be drawn from both studies, neurohistological understandings of the cerebral cortex (entorhinal and auditory) are significant. Conversely, Sacchini et al. focus the attention on the amygdaloid body of three different species, belonging to the family *Delphinidae* [striped dolphin (*Stenella coeruleoalba*), common dolphin (*Delphinus delphis*), and Atlantic spotted dolphin (*Stenella frontalis*)]. Through the use of calbindin-D28k immunohistochemistry, the authors emphasize the structure of the its central nucleus in the overall description of the amygdaloid body. The manuscript does not address the involvement of the amygdala in neurodegenerative processes, but it is well recognized in human medicine that these processes have an impact on the amygdaloid body and have been also demonstrated in one species of cetacean, the harbor porpoise (Garamszegi et al., 2024). Research on fish models has revealed additional significant facets of the neuropathological mechanisms impacting cetaceans and marine mammals overall. Po Lai et al. provide more light on the ways that hypoxia in marine medaka fish (*Oryzias melastigma*) disrupts neural function at the protein level. Hypoxia has been proposed has potential risk factor for neurodegenerative changes in the brains of cetaceans (Sacchini et al., 2020). Bernal et al. investigated the effects of ocean warming on the brain of the coral reef fish spiny chromis (*Acanthochromis polyacanthus*), assessing the biological responses that are linked to exposure to high temperatures during development and across generations: protein folding, apoptosis and cell death, modification of cellular structure, mitochondrial activity, among others. Because of ocean and global warming, harmful algal blooms are occurring more frequently in the water. In fact, cyanobacteria blooms in aquatic environments are thought to be caused by two major factors: eutrophication and climate change. Powerful cyanotoxins (BMAA), which affect both aquatic and terrestrial life, are produced by cyanobacteria. Given its association with organ system damage and disease, cyanotoxin exposures are a public health concern. As previously shown, BMAA overexposure was associated with β-amyloid+ plaques, dystrophic neurites, and TDP-43 proteinopathy in the brain of stranded dolphins, resembling AD-like pathology (Davis et al., 2019; Garamszegi et al., 2024). Lastly, Liu et al. studied two characteristic genes subtypes, in the large yellow croaker (*Larimichthys crocea*) genome, of the corticotropin-releasing hormone, the neuropeptide in the hypothalamus that is most important for the stress response. In fact, through regulating the secretion of the hypothalamic corticotropin releasing hormone, the central nucleus of the amygdala regulates the physiological and behavioral responses associated with fear and anxiety through pituitary-adrenal responses. Additionally, some species of marine mammals have been found to produce this hormone. Undoubtedly, the majority of investigations on marine mammals performed to date have clearly focused mostly on the neuropathology of inflammatory brain diseases and the neuroanatomy of the cerebral cortex (Figure 1). Gaining a solid comprehension of neuroanatomical structures is essential to improve our understanding of neuropathological/neurodegenerative processes. The next objective is to introduce a second edition of this Research Topic, which broadens the study topics and research domains in the area of neuroscience in marine mammals.

**Figure 1:**
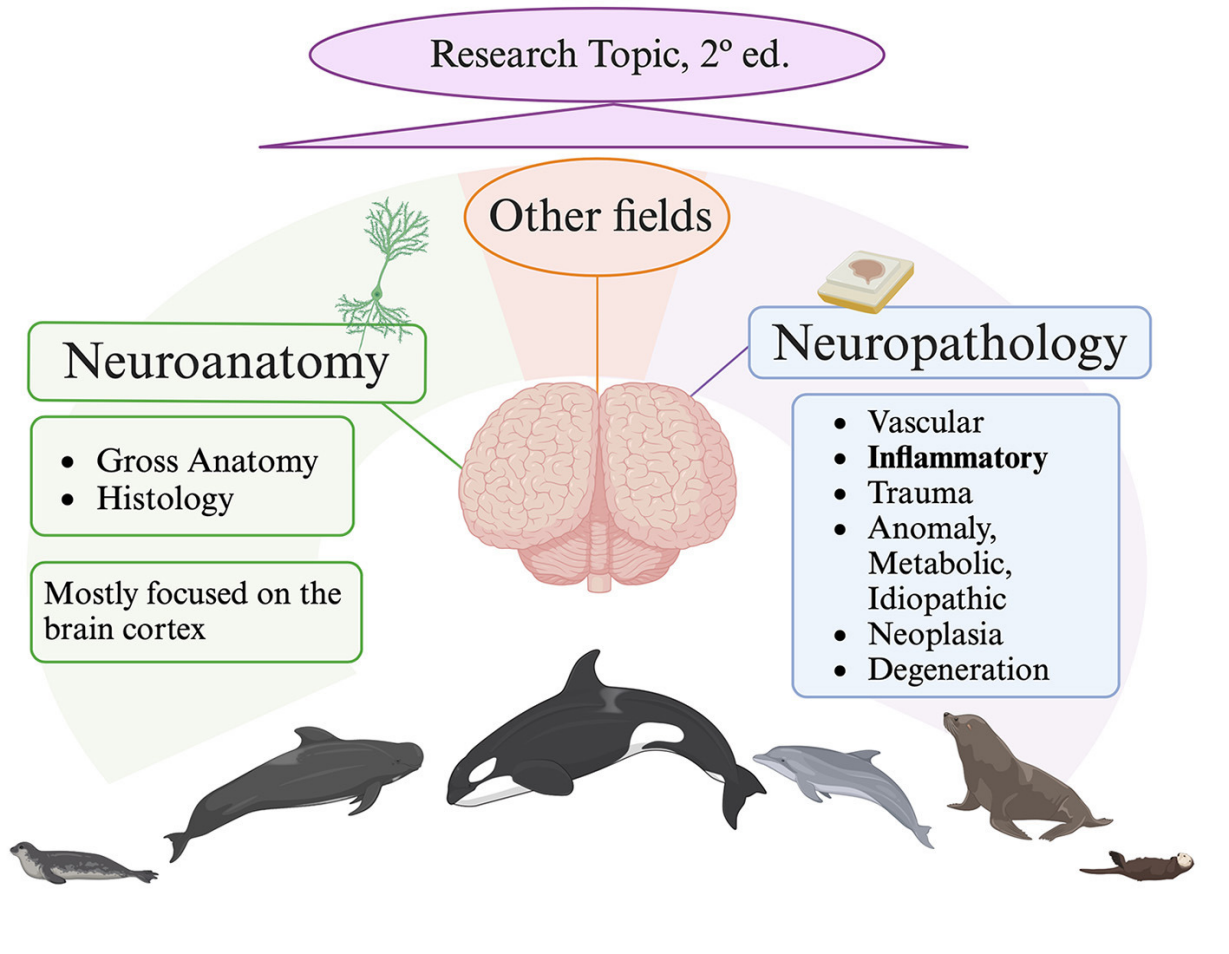
Current situation on marine mammals nervous system research. Created with Biorender.com, lastly accessed on 7 June 2024.

## Author contributions

SS: Writing – original draft, Writing – review & editing. CB: Writing – original draft, Writing – review & editing.
